# How to manage an unusual presentation of a thyroglossal duct cyst?

**DOI:** 10.1002/ccr3.3322

**Published:** 2020-09-17

**Authors:** Mafalda da Silva Ferreira, Jorge Eva Miguéis, Ana Margarida Amorim, Nuno Dias‐Silva

**Affiliations:** ^1^ ENT Department Coimbra University Hospital Coimbra Portugal; ^2^ Oncology Unit ENT Department Coimbra University Hospital Coimbra Portugal

**Keywords:** Lingual thyroglossal duct cyst, Sistrunk procedure, thyroglossal duct cyst

## Abstract

Lingual thyroglossal duct cysts are rare congenital anomalies of the neck. They can be accidentally detected or manifest with disabling symptoms. These cysts can be potentially difficult to manage, but their complete resection is curative.

## Graphical abstract

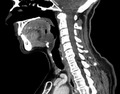

Lingual thyroglossal duct cysts (L‐TGDC) are rare congenital anomalies of the neck and comprise 0.6%‐3% of all the thyroglossal duct cysts.^1^ They are caused by retention of an epithelial tract formed during embryogenesis.^2^ Thyroglossal duct cysts usually present as a midline neck mass. L‐TGDC may be incidentally detected or manifest with disabling symptoms. Due to their location, may manifest with symptoms such as dysphagia, airway obstruction, and obstructive sleep apnea. A 62‐year‐old female patient went to a consultation complaining of a foreign body sensation in the throat since the last year. Clinical examination revealed a midline mass in the posterior part of the tongue without extraoral swelling or lymphadenopathy (Figure 1). Computed tomography scan identified a homogeneous 22 × 18 mm nodular formation in the posterior region of the base of the tongue (Figures 2 and 3). A Sistrunk procedure was performed due to the mass size. However, less invasive surgical approaches should be considered, such as transoral endoscopic‐assisted or transoral robotic excision. The histological analysis confirmed the diagnosis of L‐TGDC. After 1‐year follow‐up, the patient remains asymptomatic and has no recurrence of the mass (Figure 4). L‐TGDC can be potentially difficult to manage but their complete resection is curative.

**FIGURE 1:**
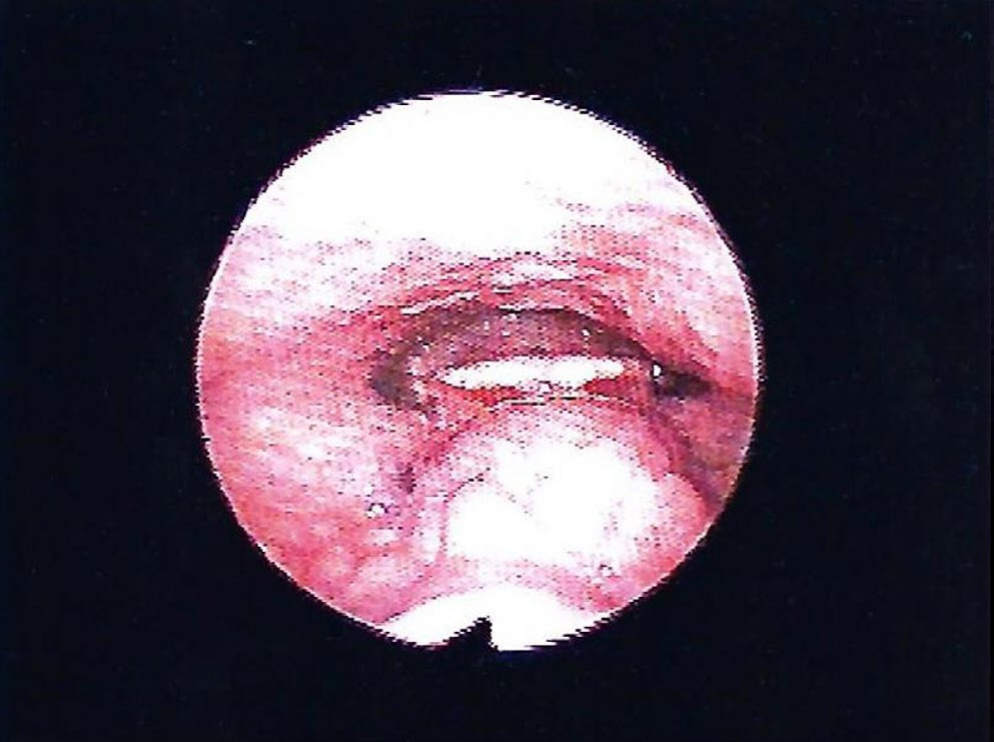
Flexible nasopharyngolaryngoscopy—midline mass in the base of the tongue

**FIGURE 2:**
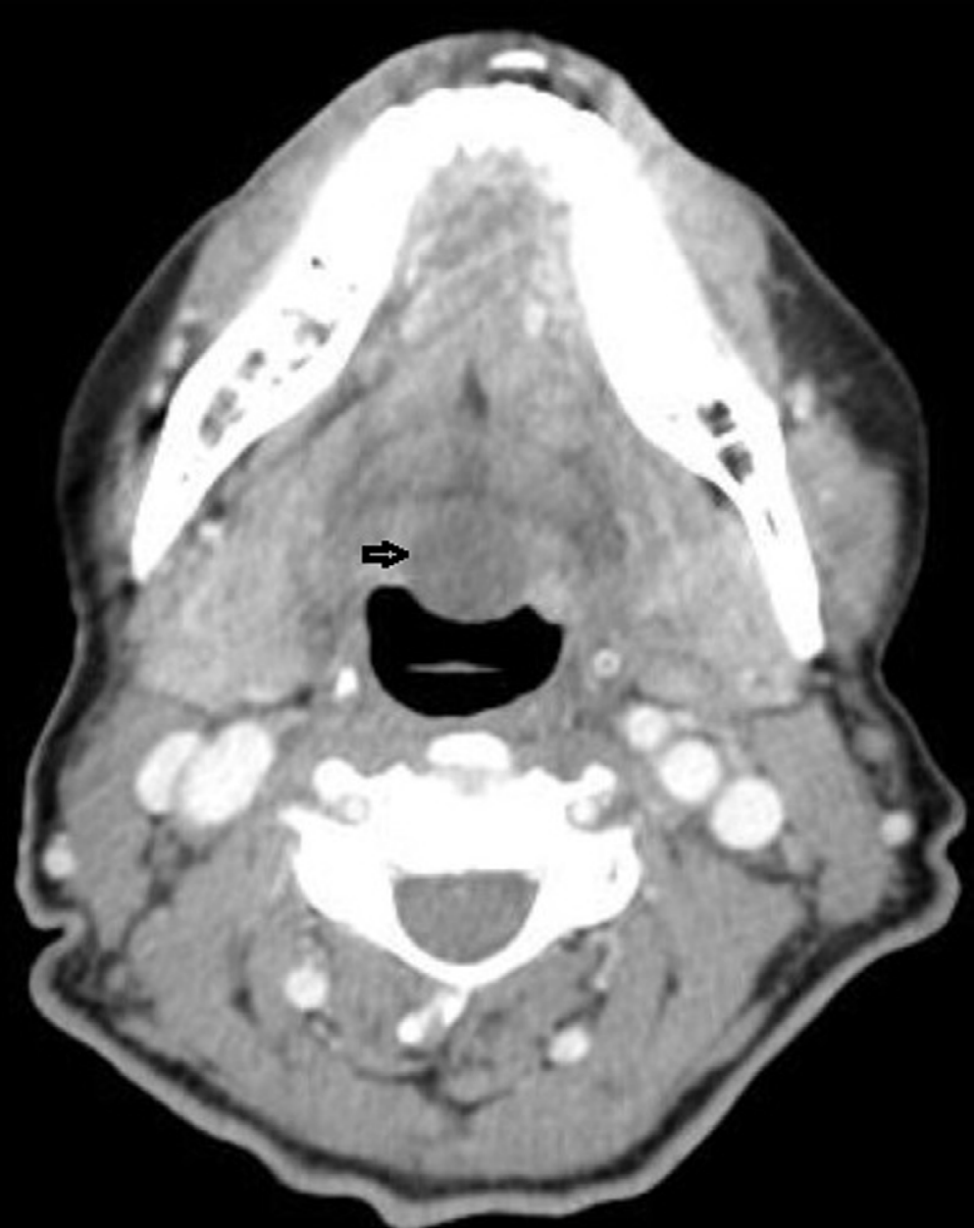
CT scan (axial plane)—homogeneous 22 × 18 mm nodular formation in the posterior region of the base of the tongue

**FIGURE 3:**
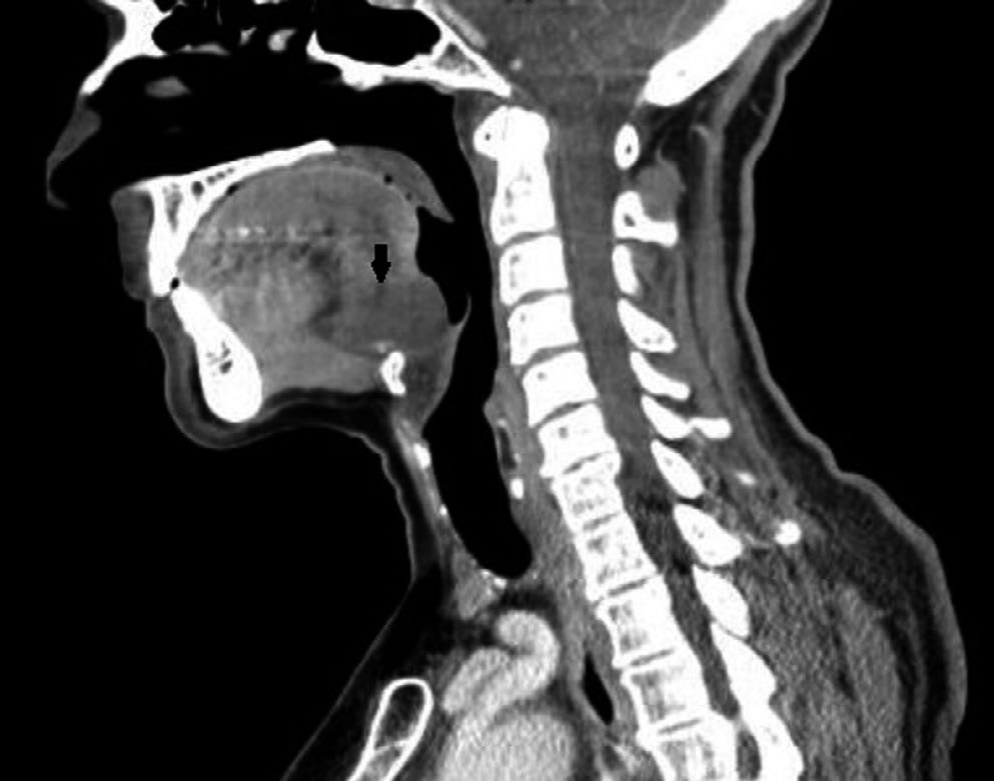
CT scan (sagittal plane)—homogeneous 22 × 18 mm nodular formation in the posterior region of the base of the tongue

**FIGURE 4:**
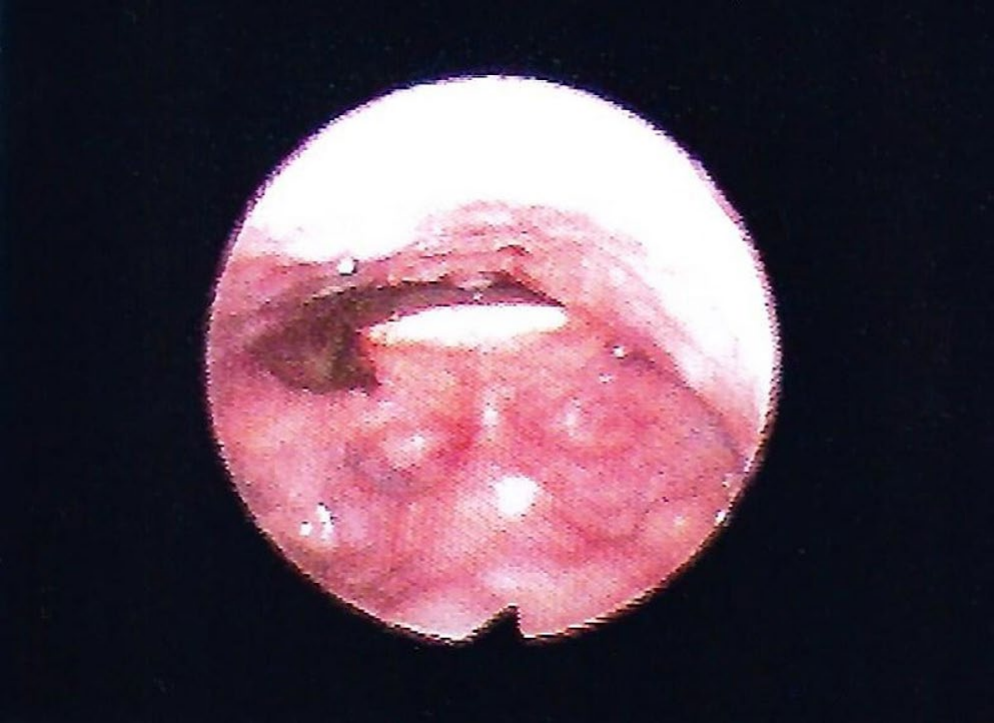
Flexible nasopharyngolaryngoscopy 1‐y follow‐up—tumefaction's absence

## CONFLICT OF INTEREST

None declared.

## AUTHOR CONTRIBUTIONS

MdSF: revised the literature and drafted the work. NS: revised the literature. AMA: involved in acquisition of clinical data and revised the work. JM (Main surgeon): revised the work and gave final approval of the version to be published.
